# Improving the Breath-Holding CVR Measurement Using the Multiband Multi-Echo EPI Sequence

**DOI:** 10.3389/fphys.2021.619714

**Published:** 2021-02-26

**Authors:** Alexander D. Cohen, Amritpal S. Jagra, Nicholas J. Visser, Baolian Yang, Brice Fernandez, Suchandrima Banerjee, Yang Wang

**Affiliations:** ^1^Department of Radiology, Medical College of Wisconsin, Milwaukee, WI, United States; ^2^Lake Erie College of Osteopathic Medicine, Erie, PA, United States; ^3^GE Healthcare, Waukesha, WI, United States; ^4^GE Healthcare, Buc, France; ^5^GE Healthcare, Menlo Park, CA, United States

**Keywords:** cerebrovascular reactivity, multiband, multi-echo, blood oxygen level-dependent, functional MRI, breath-holding

## Abstract

Blood oxygen level-dependent (BOLD) functional MRI (fMRI) is commonly used to measure cerebrovascular reactivity (CVR), which can convey insightful information about neurovascular health. Breath-holding (BH) has been shown to be a practical vasodilatory stimulus for measuring CVR in clinical settings. The conventional BOLD fMRI approach has some limitations, however, such as susceptibility-induced signal dropout at air tissue interfaces and low BOLD sensitivity especially in areas of low $T_{2}^{*}$. These drawbacks can potentially be mitigated with multi-echo sequences, which acquire several images at different echo times in one shot. When combined with multiband techniques, high temporal resolution images can be acquired. This study compared an advanced multiband multi-echo (MBME) echo planar imaging (EPI) sequence with an existing multiband single-echo (MB) sequence to evaluate the repeatability and sensitivity of BH activation and CVR mapping. Images were acquired from 28 healthy volunteers, of which 18 returned for repeat imaging. Both MBME and MB data were pre-processed using both standard and advanced denoising techniques. The MBME data was further processed by combining echoes using a $T_{2}^{*}$-weighted approach and denoising using multi-echo independent component analysis. BH activation was calculated using a general linear model and the respiration response function. CVR was computed as the percent change related to the activation. To account for differences in CVR related to TE, relative CVR (rCVR) was computed and normalized to the mean gray matter CVR. Test–retest metrics were assessed with the Dice coefficient, rCVR difference, within subject coefficient of variation, and the intraclass correlation coefficient. Our findings demonstrate that rCVR for MBME scans were significantly higher than for MB scans across most of the gray matter. In areas of high susceptibility-induced signal dropout, however, MBME rCVR was significantly less than MB rCVR due to artifactually high rCVR for MB scans in these regions. MBME rCVR showed improved test–retest metrics compared with MB. Overall, the MBME sequence displayed superior BOLD sensitivity, improved specificity in areas of signal dropout on MBME scans, enhanced reliability, and reduced variability across subjects compared with MB acquisitions. Our results suggest that the MBME EPI sequence is a promising tool for imaging CVR.

## Introduction

Blood oxygen level-dependent (BOLD) functional MRI (fMRI) is commonly used to derive cerebrovascular reactivity (CVR), an assessment of the responsiveness of blood vessels in the brain to vasoactive stimuli. CVR is an emerging marker for vascular health that can be affected by normal aging as well as neurogenerative and cognitive pathologies (Kalaria, 1996; Gorelick et al., 2011; Sweeney et al., 2019). Frequently, hypercapnic gas inhalation challenges have been performed to change the arterial carbon dioxide levels for the measurement of CVR (Tancredi and Hoge, 2013). However, the breath-holding (BH) approach also has been shown to be reliable and produces comparable CVR values (Kastrup et al., 2001; Kannurpatti and Biswal, 2008; Urback et al., 2017). Importantly, it does not require the extra equipment needed for gas inhalation, which is not available in clinical settings at all institutions (Liu et al., 2019).

Blood oxygen level-dependent fMRI is a non-invasive technique that can indirectly measure neuronal activity in the brain. BH BOLD fMRI can detect regional CVR, which is used for diagnosing and monitoring vascular changes over time. Previous studies have demonstrated regional differences in CVR that tend to vary on an individual basis (Yezhuvath et al., 2009; Catchlove et al., 2018). This intrasubject variation can be due to age or disease-related physiological changes such as vascular stiffness or metabolic responses (Ances et al., 2009). Therefore, it is important to understand the normal inter- and intra-subject variability to determine what constitutes a significant change between subjects or in the same subject over time.

It is important to note that some inherent limitations come with the BH BOLD fMRI method that need to be considered for optimizing the imaging and analysis process. For example, echo planar imaging (EPI) fMRI techniques are affected by spatial distortion and signal dropout especially in regions such as the orbitofrontal cortex (OFC), lateral parietal cortex, and inferior temporal cortex (Cho and Ro, 1992; Glover, 2011). The BH technique itself is particularly prone to lowered fMRI sensitivity due to subject motion, cardiac and respiratory cycles, and lack of compliance (Murphy et al., 2013). Intra-group variation or small effect sizes can also limit the statistical power for group analysis, particularly for patient populations (Button et al., 2013; Cremers et al., 2017).

Some of these limitations can be mitigated using advanced imaging techniques. Recent studies have shown promising outcomes using multiband EPI. By simultaneously exciting multiple slices, slice thickness can be decreased and/or temporal resolution can be increased (Posse, 2012; Kundu et al., 2013; Xu et al., 2013; Ravi et al., 2016a). In-plane spatial resolution can be further increased while maintaining reasonable TEs with in-plane acceleration techniques. Furthermore, if slice thickness is decreased, signal dropout due to susceptibility artifacts can be reduced (Schmiedeskamp et al., 2010). Multi-echo (ME) techniques, which acquire several echoes in a single excitation, have been shown to increase the sensitivity of BOLD (Posse et al., 1999; Weiskopf et al., 2005). Poser and Norris (2009) demonstrated that the signal dropout and image distortion are diminished in ME data with shorter-TE images. Multi-echo acquisitions including short TEs can optimize the BOLD contrast especially for regions known for magnetic field inhomogeneity and varying $T_{2}^{*}$ values (Posse et al., 1998; Bright and Murphy, 2013b; Ravi et al., 2016b; Fernandez et al., 2017). Boyacioglu et al. (2015) have examined the effect of adding multiband to a conventional sequence ME EPI at 7 Tesla. Their findings highlighted an increase in specificity and sensitivity as well as better resting-state network detection. This is despite the fact that decreasing $T_{2}^{*}$ with increasing field strength reduces the benefits of multi-echo acquisitions at high field strengths. Merging multiband EPI with the ME sequence can potentially harness the distinct benefits of each technique and improve some common obstacles with BOLD fMRI.

Additionally, recent studies have shown that multiband multi-echo (MBME) sequences can improve the calculation of BH metrics. In particular, the combined echo data from a simultaneous arterial spin labeling (ASL)/BOLD sequence was compared with a single echo (SE) from the same scan (Cohen and Wang, 2019). The combined echo approach produced higher BH activation and repeatability of activation and CVR compared with the single echo approach (Cohen and Wang, 2019). This sequence has also shown increased resting-state connectivity strength and volume as well as finger tapping task activation strength and volume for the combined echo approach compared with a single echo approach (Cohen et al., 2017, 2018).

These prior studies have some drawbacks. First, the study by Cohen et al. included an ASL module in Cohen and Wang (2019). This led to the repetition time (TR) being very long (4 s). Second, the “single echo” case was simply the second echo from the same multi-echo scan. Each additional echo adds to the readout time and, as a result, the TR. Because the single echo approach used the second echo from the same scan, it did not take advantage of the potential for a reduced TR. Therefore, the overall temporal resolution was reduced, which resulted in limited statistical power and inhibited a true comparison to the ME sequence.

To address these drawbacks, in this study, BOLD CVR measurements were compared between a MBME sequence and a separate multiband single-echo (MB) sequence. With a shorter TR for MB scans, the effects of multiple echoes were more directly assessed using a pure BOLD fMRI acquisition. Recent studies have also shown that relative CVR (rCVR) is a more sensitive biomarker than absolute CVR for clinical applications for both control and patient population studies (Yezhuvath et al., 2009; Pillai and Zaca, 2012; Coverdale et al., 2016). This approach also mitigates the differences in percent signal change (PSC) caused by different effective TEs for MBME and MB sequences. Thus, in this study, rCVR was compared between MBME and MB sequences. Repeatability was analyzed in a subset of subjects who returned for repeat scans. The rCVR difference between repeated scans, within subject coefficient of variation (wCV), Dice coefficient (DC) of activation, and intraclass correlation coefficient (ICC) were analyzed.

## Materials and Methods

All subjects provided written informed consent prior to participation in this study. This study was approved by the local Institutional Review Board and was conducted in accordance with the Declaration of Helsinki. In total, 28 healthy volunteer subjects (mean age = 28.0 years, range = 20–46 years, including 9 male and 19 female) participated in this study. Of them, 18 returned within 2 weeks to repeat the study for a total of 46 imaging sessions. Subjects were instructed to refrain from caffeine and tobacco for 6 h prior to imaging.

### Imaging

Imaging was performed on a 3T scanner (Signa Premier, GE Healthcare, Waukesha, WI, United States) with a body transmit coil and a 32-channel NOVA (Nova Medical, Wilmington, MA, United States) receive head coil. A 3D T1-weighted magnetization-prepared rapid acquisition with gradient echo (MPRAGE) anatomical image was acquired with TR/echo time (TE) = 2200/2.8 ms, field of view (FOV) = 24 cm, matrix size = 512 × 512 × 256, slice thickness = 0.5 mm, voxel size = 0.47 × 0.47 × 0.5 mm, and flip angle (FA) = 8°. Each subject then underwent two task fMRI scans, one MB scan, and one MBME scan. The MB scan had the following parameters: TR/TE = 650/30 ms, FOV = 24 cm, matrix size = 80 × 80, slice thickness = 3 mm (3 × 3 × 3 mm voxel size), 11 slices with multiband factor = 4 (44 total slices), FA = 60°, BW = 250 kHz, echo spacing = 0.51 ms and readout duration per excitation = 17.4 ms. Overall, 70% of total sampling time was ramp sampling. The MBME scan had the following parameters: TR = 900 ms, TE = 11,30,49 ms (three echoes), FOV = 24 cm, matrix size = 80 × 80, slice thickness = 3 mm (3 × 3 × 3 mm voxel size), 11 slices with multiband factor = 4 (44 total slices), FA = 60°, BW = 250 kHz, echo spacing = 0.51 ms, readout duration per excitation = 52.2 ms. Overall, 70% of total sampling time was ramp sampling. Both scans had partial Fourier factor = 0.85 and in-plane acceleration with *R* = 2. The functional scans lasted 320 s each for a total of 492 volumes for the MB scans and 355 volumes for the MBME scans. The TEs for the MBME scan were set to the minimum possible. The TE for the MB scan was chosen to match the second TE from the MBME acquisition.

During the functional scans, a BH task was employed. Scans began with 66 s of paced breathing, followed by four cycles of 24 s of paced breathing, 16 s of BH on expiration, and 16 s of self-paced recovery breathing. Scans ended with an additional 30 s of paced breathing. The paced breathing portions consisted of alternating 3 s inspiration and expiration blocks and were controlled using a red bar that filled up during inspiration and emptied during expiration. Participants’ breathing was monitored with a respiratory bellow to ensure task compliance. Due to time constraints, three of the repeat subjects only underwent the MBME acquisition. Two participants were excluded because they failed to complete the task for both the MBME and MB scans. An additional two subjects’ MB scans and one subject’s MBME scan were excluded due to failure to complete the task. Overall, there were 39 usable MB imaging sessions (13 repeat) and 43 usable MBME imaging sessions (17 repeat).

### SNR and CNR Calculations

All parameters for the MB and MBME data were identical except for the number of echoes and TR. Thus, the signal intensity was estimated for the MB and MBME data to evaluate the effects of FA and TR on SNR using Eq. 1. Here, T1 is the T1 of gray matter at 3T, estimated to be 1.3 s. The ratio of S between the MBME and MB sequences was then computed.

(1) $$S=\frac{\sin\left(\text{FA}\right)\cdot \left(1-e^{-\frac{T\,R}{T\,1}}\right)}{1-\cos\left(\text{FA}\right)\cdot e^{-\frac{T\,R}{T\,1}}}$$

For the single echo case the contrast to noise ratio (CNR) was estimated using Eq. 2 (Posse et al., 1999; Poser et al., 2006; Miletic et al., 2020) where S_0_ is the signal at TE = 0 ms and σ_0_ is the noise.

(2) $$C\,N\,R_{M\,B}\,\left(T\,E\right)=\frac{S_{0}}{\sigma_{0}}\cdot T\,E\cdot e^{-\frac{T\,E}{T_{2}^{*}}}$$

For the multi-echo case, the CNR was estimated using Eq. 3 (Poser et al., 2006; Miletic et al., 2020) for the $T_{2}^{*}$-weighted echo combination approach used below.

(3) $$C\,N\,R_{M\,B\,M\,E}\,\left(T\,E\right)=\frac{\sum_{n=1}^{N}W_{n}\cdot T\,E_{n}\cdot \frac{S_{n}}{\sigma }}{\sqrt{\sum_{n-1}^{N}W_{n}^{2}}}$$

Here, *n* is the echo number, *N* is the total number of echoes, *S*_*n*_ is the signal intensity at each echo estimated by:

(4) $$S_{n}=e^{-\frac{T\,E_{n}}{T_{2}^{*}}}$$

and *W*_*n*_ are the weighting factors given by:

(5) $$W_{n}=\frac{T\,E_{n}\cdot e^{-\frac{T\,E}{T_{2}^{*}}}}{\sum_{n=1}^{N}T\,E_{n}\cdot e^{-\frac{T\,E}{T_{2}^{*}}}}$$

The ratio of CNR_*MBME*_ to CNR_*MB*_ was then evaluated at $T_{2}^{*}$ = 50 ms, a typical value of $T_{2}^{*}$ in gray matter at 3T (Wansapura et al., 1999).

### Pre-processing

Data was analyzed using a combination of AFNI (Cox, 1996), FSL (Jenkinson et al., 2012), and MATLAB (The Mathworks, R2018a). Image pre-processing utilized the Human Connectome Project (HCP) minimal pre-processing pipeline (Glasser et al., 2013), modified to account for the multiple echo data (Cohen and Wang, 2019).

Anatomical processing was completed using the PreFreeSurferPipeline.sh scripts from the HCP pipeline. First, the anatomical MPRAGE image was anterior commissure–posterior commissure (ACPC) aligned using *aff2rigid* in FSL, and a brain mask was created using FNIRT-based brain extraction as follows. First, the MPRAGE images was linearly registered to MNI space using *flirt* in FSL with 12 degrees of freedom (Jenkinson and Smith, 2001; Jenkinson, 2002). Then, *fnirt* in FSL was used to non-linearly refine the registration (Andersson et al., 2007). A brain-only reference image in MNI space was then inverse warped to native space using the transformations determined by the *flirt and fnirt* steps and used to extract the brain. The brain-only MPRAGE image was then registered to Montreal Neurological Institute (MNI) space using *flirt* with 12 degrees of freedom (Jenkinson, 2002) to linearly register the MPRAGE image to MNI space followed by *fnirt* to non-linearly refine the registration.

For both the MB and MBME datasets, the first 10 volumes were discarded to allow the signal to reach equilibrium. Each dataset was then volume registered to the first volume using *mcflirt* in FSL. For the MBME data, only the first-echo dataset was registered. Subsequent echoes were registered using the transformation matrices from the first echo. Then, the three echoes were combined using the $T_{2}^{*}$-weighted approach (Posse et al., 1999).

The analysis pipeline then split into two separate analyses. First the MB and MBME data were processed identically using “standard” denoising procedures. Then, the MB and MBME data were reprocessed using “advanced” denoising procedures: an independent component analysis-based strategy for Automatic Removal of Motion Artifacts (ICA-AROMA) (Pruim et al., 2015; Dipasquale et al., 2017) and multi-echo independent component analysis (ME-ICA), respectively.

The standard denoising pipeline consisted of first, registering the MB and MBME datasets to the ACPC-aligned MPRAGE image using *epi_reg* in FSL and then registering both datasets to MNI space using the anatomical transformations computed above. The data was smoothed using a 4 mm full width at half maximum (FWHM) Gaussian kernel and detrended with a third-order polynomial. The six rigid-body motion parameters were regressed from the data.

The advanced denoising pipeline for the MB data consisted of first, registration to the ACPC-aligned MPRAGE image using *epi_reg* in FSL and then to MNI space using the anatomical transformations computed above. The data was smoothed using a 4 mm full width at half maximum (FWHM) Gaussian kernel and then denoised using an ICA-AROMA (Pruim et al., 2015; Dipasquale et al., 2017). ICA-AROMA is a data-driven technique which removes components related to motion from the data. The six rigid-body motion parameters were regressed from the data.

The advanced denoising pipeline for the MBME data utilized ME-ICA and the open source python script tedana.py version 0.0.9^1^ (Kundu et al., 2012, 2013; DuPre et al., 2019). This technique, described in detail elsewhere, classifies independent components as BOLD or non-BOLD based on whether or not their amplitudes are linearly dependent on TE, respectively (Kundu et al., 2012, 2013; Olafsson et al., 2015). Non-BOLD components were regressed out of the combined ME data resulting in a denoised dataset. The denoised MBME dataset was then registered to the ACPC-aligned MPRAGE image using *epi_reg* in FSL, and subsequently registered to MNI space using the anatomical transformations computed above. Finally, the data was smoothed using a 4 mm FWHM Gaussian kernel. No additional nuisance regressors were removed from the MBME data.

A significant problem arose using both ICA-AROMA and ME-ICA to process the BH data. For most of the datasets, the BH response was erroneously classified as non-BOLD and regressed from the data. For the ME-ICA technique this was due to the high variance of the BH response, which caused the signal to be classified as noise despite a strong dependence on TE. Removing the variance criteria from the algorithm caused true high-variance noise components to be falsely classified as BOLD. Therefore, an additional pre-processing step was added for the MBME data wherein the task frequency (*f* = 1/56 s) was bandpass filtered out of the data. ICA-AROMA and ME-ICA were then run on the filtered data, and the noise components were regressed out of the original, unfiltered dataset. This eliminated the problem of the task response being regressed from the data.

The temporal signal-to-noise ratio (tSNR) was computed after the smoothing step by dividing the voxelwise mean signal across time by the standard deviation of the time series. Mean voxelwise tSNR across subjects for each scan was calculated. To account for differing numbers of time points between the MBME and MB scans, the normalized tSNR, tSNR_*norm*_, was computed by multiplying tSNR by $\sqrt{N_{T\,P}}$ where *N*_*TP*_ is the number of time points (Smith et al., 2013).

### fMRI Processing and BH Response Analysis

The BH response was evaluated in an identical fashion for the standard and advanced denoising techniques by using a general linear model with *3dDeconvolve* in AFNI. After *3dDeconvolve*, a restricted maximum likelihood model (*3dREMLfit*) was used to model temporal autocorrelations in the data. This program uses an ARMA(1,1) to model the time series noise correlation in each voxel. BH regressors were generated by convolving a square wave, with ones during BH periods and zeros otherwise, with the respiration response function (Eq. 6) (Birn et al., 2008). The BH hemodynamic response is slow, with the peak oftentimes occurring after the BH period. Thus, most studies time shift the BH regressor by several seconds to better model the response (Kastrup et al., 1999b; Birn et al., 2008; Magon et al., 2009). While the respiration response function implicitly takes this delay into account, the BH response delay also varies across the brain by as much as ±8 s (Birn et al., 2008; Bright et al., 2009; Bright and Murphy, 2013a; van Niftrik et al., 2016). To account for this, the BH regressor was shifted from -8 to 16 s in steps of 2 s, and for each voxel, the regressor that resulted in the highest positive *t*-score was chosen.

(6) $$R\,R\,F\,\left(t\right)=0.6\cdot t^{2.1}\,e^{-t\,/\,1.6}-0.0023\cdot t^{3.54}\,e^{-t\,/\,4.25}$$

Both the ICA-AROMA and ME-ICA techniques involve regressing noise components from the data that differ across technique and subject. Therefore, the number of degrees of freedom was adjusted based on the number of components regressed from the data by the ICA-AROMA or ME-ICA steps for the MB and MBME data, respectively. The *t*-scores were then converted to *z*-scores, which were corrected for the reduced degrees of freedom.

### CVR Calculation

Cerebrovascular reactivity was calculated for the MB and MBME data as the PSC of the BH task response. This was computed by dividing the beta coefficient of the BH response by the mean signal. The PSC increases with TE (Fujita, 2001; Yacoub et al., 2003; Triantafyllou et al., 2011). Because the combined MBME data is a weighted average with shorter TEs, the PSC tended to be lower than for the MB data. Thus, to account for differences in CVR related to the TE, rCVR was computed as the CVR divided by the mean CVR in gray matter (Yezhuvath et al., 2009; Pillai and Zaca, 2012; Coverdale et al., 2016).

### Test–Retest Analysis

The similarity of BH activation across time was assessed using the Dice Coefficient (DC) (Eq. 7). The DC measures the degree of overlap of active voxels between scans acquired at different times. In this equation, OV_*T1*–*T2*_ is the number of overlapping active voxels between time point 1 (TP1) and time point 2 (TP2), V_*T1*_ is the number of active voxels at TP1 and V_*T2*_ is the number of active voxels at TP2. For DC calculations, activation maps were thresholded at an uncorrected *p* < 0.01.

(7) $$D\,C=\frac{2\cdot O\,V_{T\,1-T\,2}}{V_{T\,1}+V_{T\,2}}$$

The voxelwise repeatability of rCVR was evaluated using the difference between rCVR at TP1 and TP2 and the within subject coefficient of variation (wCV), defined as the standard deviation divided by the mean across the two TPs. The rCVR difference and wCV were then averaged across gray matter (GM).

Lastly, voxelwise ICC(3,1) was calculated to analyze the test–retest reliability. ICC(3,1) ranges from 0 to 1, where a value of 1 expresses perfect reliability. Voxelwise ICC was calculated using *3dICC* in AFNI (Chen et al., 2018). Although there are no standard ranges indicating what is “good” reliability, a general recommendation categorizes ICC less than 0.4 as poor, between 0.4 and 0.6 as fair, between 0.6 and 0.8 as good, and greater than 0.8 as excellent (Cicchetti, 1994). In accordance with these parameters, the ICC maps were thresholded at 0.4, 0.6, and 0.8, and the percentage of voxels above those thresholds were calculated.

### Group Comparisons

For all paired *t*-tests a *p*-value < 0.05 was considered significant. Mean whole-brain normalized tSNR was extracted, and compared between MB and MBME datasets using a paired *t*-test.

The DC, rCVR difference, and wCV were compared between MB and MBME scans using a paired *t*-test.

Both voxelwise rCVR and *z*-score were compared between MB and MBME datasets using *3dLME* in AFNI (Chen et al., 2013). *3dLME* performs a voxelwise linear mixed-effects (LME) analysis in cases where subjects have more than one measurement. It can also handle missing data. General linear tests (GLTs) were included to compute group rCVR and activation *z*-score measurements for MB, MBME, and the difference between MBME and MB datasets. Mean rCVR and *z*-score maps were also output for MBME and MB data. The residuals from the model were used as input into *3dFWHMx* in AFNI to estimate the autocorrelation function (acf) parameters. The acf parameters were then used as input into *3dClustSim* in AFNI to estimate the minimum cluster size required for a cluster to be considered significant (Cox et al., 2017). Group data were thresholded at *p* < 0.05 (cluster size corrected with minimum cluster size = 870 voxels, α < 0.05). Voxelwise group comparisons were repeated for the standard and advanced denoising pipelines.

For additional quantitative comparisons, individual mean *z*-scores were extracted both from GM (unthresholded) and from all active voxels (thresholded at *p* < 0.01). Also, the total number of active voxels were extracted for each scan. These metrics were compared between the MB and MBME datasets for both the standard and advanced denoising pipelines using paired *t*-tests.

A region-of-interest (ROI) analysis was performed where the mean rCVR value was extracted from each of 17 ROIs from the Yeo-17 network template (Yeo et al., 2011). A Bonferroni-corrected paired *t*-test compared rCVR between MB and MBME data for each ROI for both the standard and advanced denoising pipelines.

In addition, the mean time series was extracted from all GM voxels, voxels with 2 < *z* < 2.5, and voxels with *z* > 4.0 for MB and MBME datasets averaged across all subjects. To examine potential improvements with the combined-echo data in active voxels, a mask was created using the thresholded voxels in the MB data. That same mask was applied to the MBME data. Thus, the same voxels were analyzed for the MB and MBME datasets.

## Results

### SNR and CNR Calculations

**FIGURE 1:**
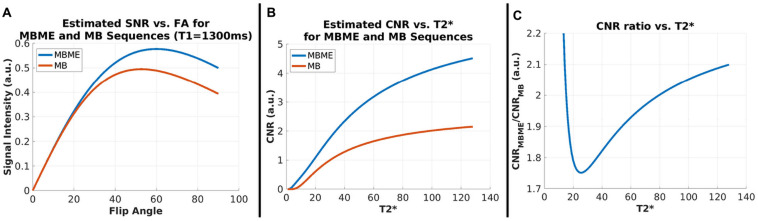
SNR and CNR Simulations. **(A)** Signal intensity vs. flip angle for MBME and MB sequences with T1 = 1300 ms. Signal for MBME data is higher owing to the longer TR (900 ms vs. 650 ms). The flip angle for both sequences was 60°. **(B)** CNR vs. $T_{2}^{*}$ for MBME and MB sequences. CNR increased with $T_{2}^{*}$ and was higher for the MBME sequences. **(C)** The CNR ratio of MBME to MB sequences vs. $T_{2}^{*}$.

The estimated signal intensity ratio between the MBME and MB scans based on TR and FA and calculated using Eq. 1 was 1.18 for T1 = 1.3 s. Thus, the higher TR and optimized FA for the MBME scans resulted in an expected 1.18× SNR advantage over the MB scans assuming noise was equivalent. Plots showing estimated signal intensity as a function of FA for the MB and MBME scans are shown in Figure 1A.

The estimated CNR ratio between MBME and MB scans, calculated using Eqs 2–5 was 1.88 for $T_{2}^{*}$ = 50 ms. Plots showing the estimated CNR as a function of $T_{2}^{*}$ and estimated CNR ratio as a function of $T_{2}^{*}$ are shown in Figures 1B,C, respectively. For $T_{2}^{*}$ values greater than approximately 25 ms the CNR ratio increased with increasing $T_{2}^{*}$.

### tSNR

**FIGURE 2:**
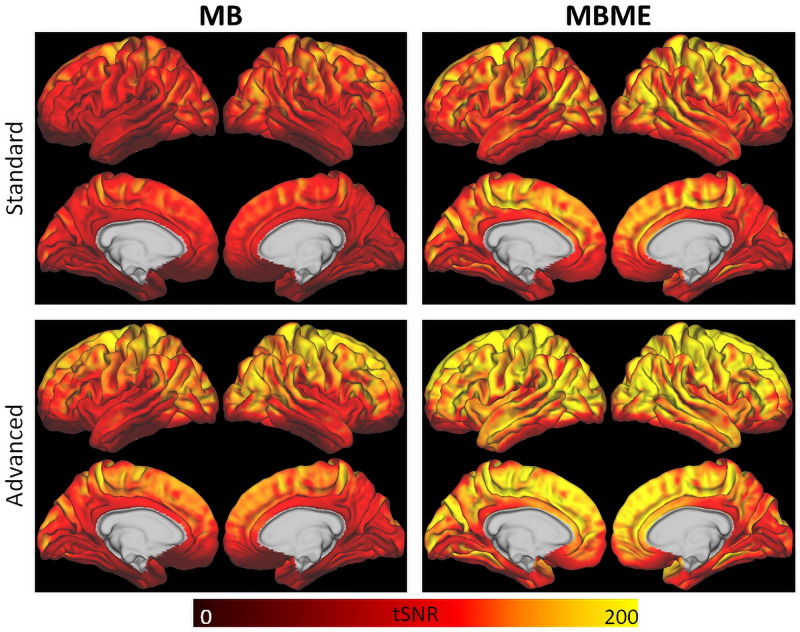
tSNR maps for the standard **(top)** and advanced **(bottom)** denoising pipelines. tSNR was higher for the advanced denoising pipeline compared to the standard denoising pipeline and for the MBME data (right) compared to the MB data (left).

For the standard denoising pipeline tSNR_*norm*_ was significantly higher for the MBME datasets compared with the MB datasets (2327 ± 358 vs. 2089 ± 292, respectively, *p* < 0.001). Similarly, for the advanced denoising pipeline normalized tSNR was significantly higher for the MBME datasets compared with the MB datasets (2784 ± 329 vs. 2500 ± 295, respectively, *p* < 0.001). Mean tSNR maps are shown in Figure 2.

### BH Activation and rCVR Comparisons

**FIGURE 3:**
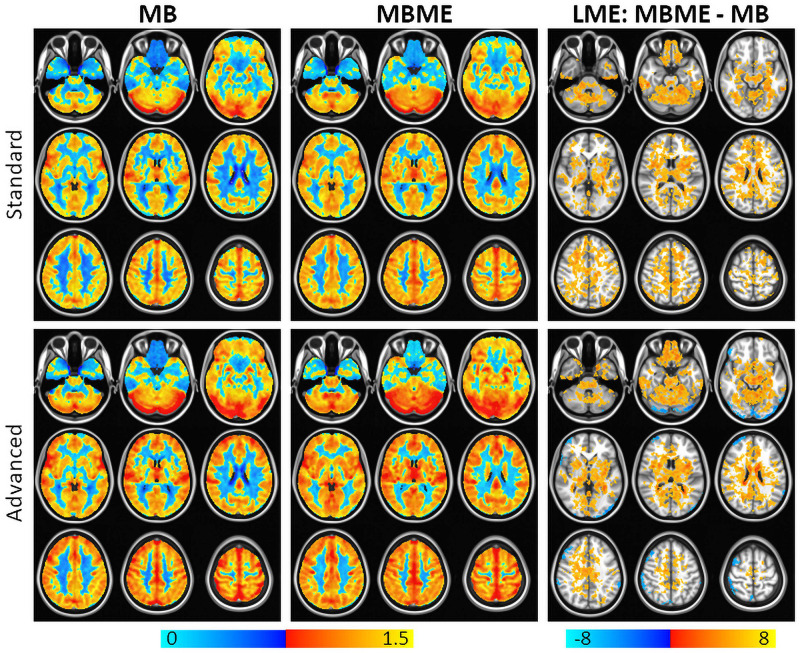
Average breath-holding activation *z*-score maps for MB (left) and MBME (middle) scans for the standard (top) and advanced (bottom) denoising pipelines. BH activation was generated from a general linear model using the respiration response function as a regressor. The right column shows a comparison of the BH activation *z*-score between MBME and MB scans using a general linear test in the frame of the mixed linear model. Maps were thresholded at a cluster-corrected *p* < 0.05 (corresponding to α < 0.05 at the cluster level). For both processing pipelines BH activation was higher for the MBME data vs. MB data. There was one cluster where MB activation was higher the MBME activation for the advanced pipeline. Orange clusters indicate significant regions where MBME > MB. Blue clusters indicate significant regions where MBME < MB.

Maps of average *z*-score and results of a GLT comparing MBME and MB *z*-scores are shown in Figure 3 for the standard and advanced denoising pipelines. Qualitatively, for both the standard and advanced denoising pipelines, mean activation was higher in GM for MBME vs. MB scans. This was confirmed by the GLT results that showed increased activation for MBME compared with MB scans. The largest clusters were in the prefrontal cortex, OFC, subcortical regions, medial temporal area, and posterior cingulate cortex. For the advanced denoising pipeline, there was one cluster with higher activation for the MB vs. MBME data located in the cerebellum and visual cortex.

**FIGURE 4:**
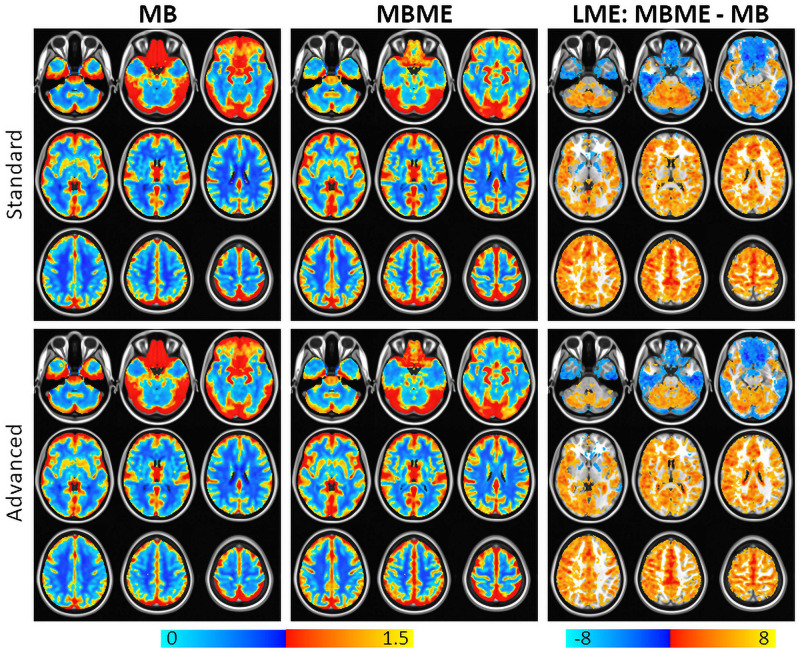
Mean rCVR for MB (left) and MBME (middle) scans for the standard (top) and advanced (bottom) denoising pipelines. The right column shows a comparison of rCVR between MBME and MB scans using a general linear test in the frame of the mixed linear model. Maps were thresholded at a cluster-corrected *p* < 0.05 (corresponding to α < 0.05 at the cluster level). For most gray matter regions, MBME scans produced significantly higher rCVR than MB scans. In the regions associated with high susceptibility induced signal dropout including the orbitofrontal cortex and inferior temporal lobe, rCVR was higher for MB scans compared to MBME scans. Results were very similar for the standard and advanced denoising pipelines. Orange clusters indicate MBME > MB. Blue clusters indicate MBME < MB.

Maps of mean rCVR and results of a GLT comparing MBME and MB rCVR are shown in Figure 4 for the standard and advanced denoising pipelines. Qualitatively, for both the standard and advanced denoising pipelines, mean rCVR was higher for MBME vs. MB scans throughout much of the GM with the exception of the OFC and inferior temporal lobes. This was confirmed by the GLT results that showed higher rCVR for MBME scans compared with MB scans throughout much of the GM. MBME rCVR was lower than MB rCVR, however, in the OFC and inferior temporal lobes, areas associated with susceptibility-induced signal dropout. tSNR was lower in these regions compared to the rest of the brain for both sequences and analyses, however less so for the MBME sequence (Figure 2). The qualitative rCVR measurements were very similar between the two sequences with the exception of the OFC and inferior temporal cortex, areas of high signal dropout. There were also global increases in tSNR for the MBME sequence compared to the MB sequence, but the GLT results showed higher rCVR in most of the gray matter and lower rCVR in high susceptibility areas.

Quantitative BH activation results are shown in Tables 1, 2 for the standard and advanced denoising pipelines, respectively. No significant difference in mean *z*-score were seen for MBME vs. MB scans for both the standard and advanced denoising pipelines; however, the total number of active voxels was higher for MBME vs. MB scans for the standard and advanced denoising pipelines.

**TABLE 1:** Comparison of breath-holding activation for MB and MBME scans using the standard processing pipeline.

	Mean *z*-score	Mean *z*-score	Number of
	in GM	Active Voxels	Active Voxels
MB	4.93 (1.25)	5.29 (0.94)	142822 (28441)
MBME	5.27 (1.20)	5.44 (0.92)	156807 (23599)
*p*-value	0.153	0.677	0.001

*Numbers in parentheses indicate standard deviation. MB, multiband single-echo; MBME, multiband multi-echo; GM, gray matter.*

**TABLE 2:** Test–retest analysis for the MB and MBME datasets using the standard processing pipeline.

	Dice	wCV	Difference	Voxelwise	% Voxels with ICC		
	Coefficient			ICC in GM	Greater Than:		
	
					0.4	0.6	0.8
MB	0.875 (0.060)	0.206 (0.043)	0.40 (0.06)	0.681 (0.223)	85.1	68.5	35.4
MBME	0.899 (0.029)	0.174 (0.023)	0.31 (0.05)	0.697 (0.213)	88.0	73.2	38.3
*p*-value	0.057	0.003	4.5E-05	N/A	N/A		

*Numbers in parentheses indicate standard deviation. MB, multiband single echo; MBME, multiband multi-echo; GM, gray matter; ICC, intraclass correlation coefficient, wCV, within subject coefficient of variation.*

**FIGURE 5:**
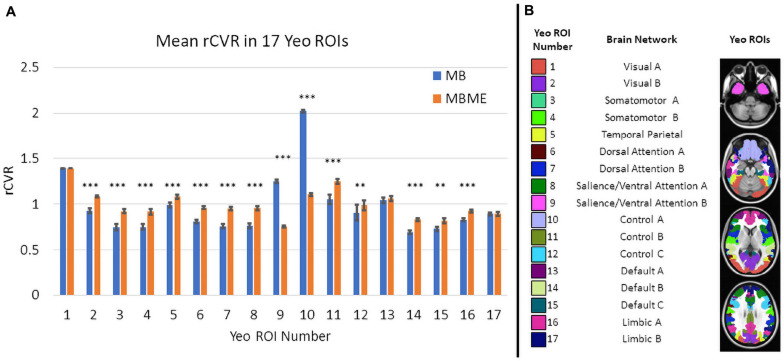
**(A)** Comparison of mean rCVR between MB and MBME scans in 17 different Yeo ROIs for the advanced denoising pipeline. Significant differences between seque01nces were found in for 14 out of 17 ROIs. There were 12 ROIs that showed MBME > MB and two ROIs where MB > MBME. The Yeo ROI 10 (the Control A network) displayed the largest difference. **(B)** The corresponding Yeo ROIs to the data in panel **(A)**. (Note: * *p* < 0.05, ** *p* < 0.01, *** *p* < 0.001, Bonferroni-corrected).

Comparisons of ROI-averaged rCVR between MB vs. MBME scans is shown in Figure 5A for the advanced denoising pipeline and mirrored the voxelwise analysis. Mean rCVR was significantly higher for MBME vs. MB data in 12 of 17 Yeo ROIs, while mean rCVR was higher for MB vs. MBME data in two Yeo ROIs (9 and 10). These two ROIs encompass large areas of susceptibility-induced signal dropout (orbitofrontal gyrus and inferior temporal lobe). In fact, the largest mean rCVR difference was observed for Yeo 10, where rCVR for MB data was significantly greater than MBME data (2.02 ± 0.55 vs. 1.11 ± 0.35, respectively; *p* < 0.0001). Corresponding Yeo ROIs are shown in Figure 5B. Yeo ROIs 9 and 10 encompass the inferior frontal and orbitofrontal cortices, areas that suffer from susceptibility induced signal dropout.

### Test–Retest Analyses

**FIGURE 6:**
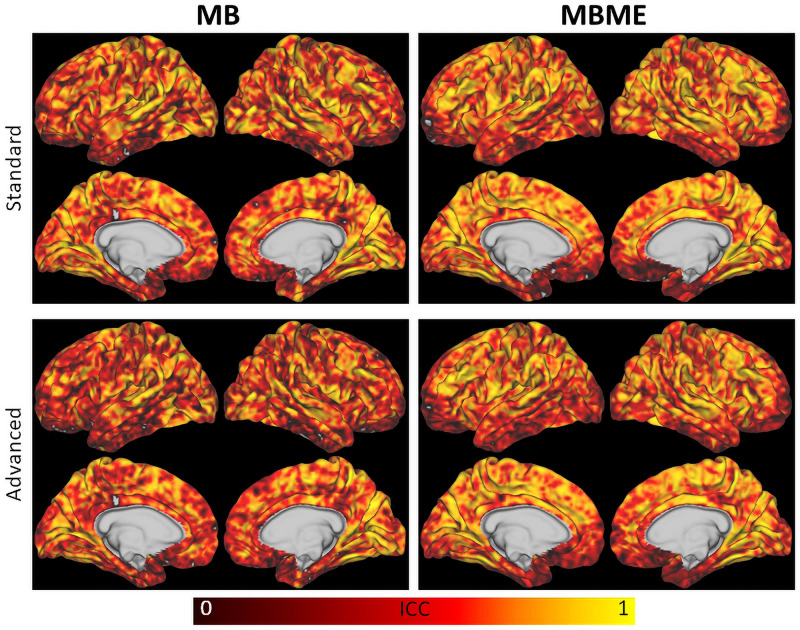
Surface maps of the intraclass correlation coefficient for MB (left) and MBME (right) scans for the standard (top) and advanced (bottom) denoising pipelines. The MBME data had higher ICC than the MB data throughout most of the brain, as indicated by more orange and yellow areas.

Results from the repeatability analysis are shown in Tables 3, 4 for the standard and advanced denoising pipelines, respectively. The MBME scans produced a higher DC than MB, but it only trended toward significance for the standard denoising pipeline (*p* = 0.057). The mean difference in rCVR between TP1 and TP2 and wCV were significantly lower (i.e., more repeatable) for MBME compared with MB scans for both pipelines. Both MB and MBME scans had “good” reliability, estimated via ICC with values ranging from 0.65 to 0.70. ICC for MBME data was higher compared with MB for both denoising pipelines as was the percentage of voxels with ICC > 0.4, 0.6, and 0.8. For example, for the advanced denoising pipeline 34.8% of voxels had ICC > 0.8 for MBME compared with 28.8% for MB data. Surface maps of voxelwise ICC are shown in Figure 6.

**TABLE 3:** Comparison of breath-holding activation for MB and MBME scans using the advanced processing pipeline.

	Mean *z*-score	Mean *z*-score	Number of
	in GM	Active Voxels	Active Voxels
MB	5.61 (1.28)	5.83 (0.96)	153924 (23132)
MBME	5.87 (1.26)	6.01 (0.98)	164843 (18042)
*p*-value	0.445	0.574	0.002

*Numbers in parentheses indicate standard deviation. MB, multiband single-echo; MBME, multiband multi-echo; GM, gray matter.*

**TABLE 4:** Test–retest analysis for the MB and MBME datasets using the advanced processing pipeline.

	Dice	wCV	Difference	Voxelwise	% Voxels with ICC		
	Coefficient			ICC in GM	Greater Than:		
	
					0.4	0.6	0.8
MB	0.889 (0.054)	0.219 (0.056)	0.42 (0.08)	0.647 (0.223)	81.2	62.3	28.8
MBME	0.907 (0.024)	0.187 (0.027)	0.33 (0.05)	0.678 (0.213)	86.5	69.7	34.8
*p*-value	0.169	0.031	3.6E-04	N/A	N/A		

*Numbers in parentheses indicate standard deviation. MB, multiband single echo; MBME, multiband multi-echo; GM, gray matter; ICC, intraclass correlation coefficient, wCV, within subject coefficient of variation.*

### Signal Quality

**FIGURE 7:**
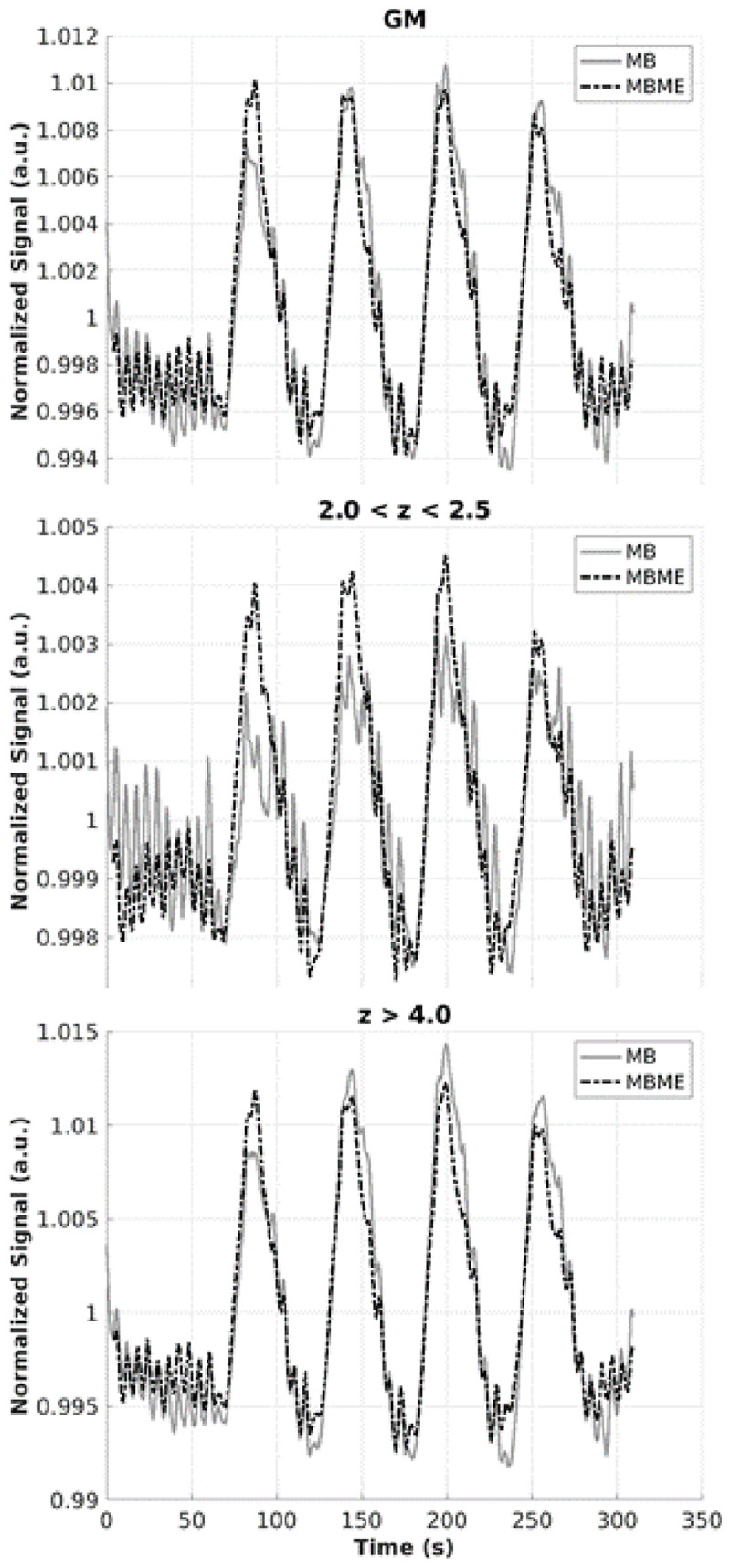
Mean time series for MB and MBME scans for gray matter **(top)**, active voxels with *z*-scores between 2 and 2.5 **(middle)**, and active voxels with *z*-score greater than 4 **(bottom)** for the advanced denoising pipeline. There was a notable improvement in signal quality for MBME vs. MB of active voxels with the threshold ranged from 2 to 2.5, while the signals for the other categories appeared similar.

The average time series for MB and MBME datasets are shown in Figure 7 for the advanced denoising pipeline. For the active voxels thresholded at 2.0 < *z* < 2.5, there was a marked increase in signal quality for the MBME data vs. MB data. For active voxels with a higher threshold of *z* > 4.0 and averaging across the whole GM (unthresholded), there was no clear difference in signal quality between the two acquisitions. Oscillations of approximately 0.17 Hz corresponding to the paced breathing were seen for all data in Figure 7. They were similar for the GM and *z* > 4 timeseries but the oscillation amplitude was higher for the MB sequence for 2 < *z* < 2.5 case.

## Discussion

In the present study, rCVR was compared between MB and MBME sequences using standard and advanced denoising techniques. The MBME sequence resulted in higher voxelwise BH activation *z*-scores and volume and improved repeatability compared with the MB sequence. Notably, the MBME approach had higher specificity in regions with high signal dropout and reduced variability across subjects. These results suggest that the MBME sequence with appropriate processing is a robust technique for reliable BH CVR measurements.

One major advantage of acquiring three echoes using the MBME sequence is that $T_{2}^{*}$ can be estimated and used as a weighting factor when combining echoes. In this technique, echoes more closely matching the estimated $T_{2}^{*}$ value are weighted higher. In this way, sensitivity can be increased as BOLD sensitivity has been shown to be higher when TE = $T_{2}^{*}$. Furthermore, acquiring more than two echoes allows for denoising with ME-ICA, where the TE dependence of BOLD signals can be measured and then separated from spurious noise signals (Kundu et al., 2012, 2013; Olafsson et al., 2015). Physiological fluctuations, including heartbeat, respiratory, and motion artifacts, in the signal can be automatically removed. This underlying benefit was reinforced with the rCVR repeatability analysis where the rCVR difference, wCV, and ICC were improved for MBME vs. MB data (Table 3).

It is also important to note that parameters were identical between the MB and MBME acquisitions with the exception of TR (900 ms for MBME and 650 ms for MB). This was deliberate as the goal was to evaluate each sequence as researchers were likely to use them. Therefore, TR was minimized for each sequence. SNR calculations showed that the higher TR for the MBME data resulted in an SNR 1.18 times higher than the MB data. This fact could potentially lead to biased results and an unfair comparison between the sequences. However, since the scan time was the same for each sequence, the MB data contained 1.39 times the number of time points compared to the MBME data (492 vs. 355, respectively). Murphy et al. (2007) have shown that the tSNR required to detect a given effect size is proportional to $1\,/\,\sqrt{N}$ where N is the number of timepoints. Thus, as N increases the required tSNR decreases. So, while the higher TR for MBME scans resulted in a 1.18 times SNR increase, it also increased the tSNR necessary to detect a given effect size. To detect the same effect size, the MBME data requires a tSNR of 1.18 times the tSNR of the MB data effectively canceling out the SNR gains related to the TR. Importantly, the fact that these values, the SNR gain caused by the longer TR and the tSNR increase required to detect the same effect size, are virtually identical is a coincidence and only effectively cancel for this specific set of parameters with T1 = 1.3 s.

Compared with the BH CVR report by Cohen and Wang (2019), this study produced markedly higher voxelwise ICCs (ICC > 0.65 vs. ICC < 0.5). Part of this may be due to the significantly increased temporal resolution in the current study compared with that in Cohen et al. (TR = 0.65–0.9 s vs. TR = 4.0 s, respectively), resulting in more than six times the number of timepoints for the MB scans compared to Cohen and Wang (2019). In addition, the study by Cohen and Wang (2019) included pCASL labeling, which may have added additional noise sources to the data.

Multiband multi-echo scans resulted in higher activation volume compared with MB scans. These results indicate a better response is obtained from MBME than MB. Furthermore, the mean time series was extracted from MB and MBME data in several regimes: unthresholded whole GM, active voxels at the threshold of 2.0 < *z* < 2.5, and active voxels at the threshold *z* > 4.0 (Figure 7). The biggest differences in the two signals occurred in the low threshold regime (2.0 < *z* < 2.5) where the MBME signal was cleaner than the MB signal. This is in accord with the results from Cohen and Wang (2019), who found significant improvements for multi-echo data compared with single echo data for voxels with 0.01 < *p* < 0.05.

Independent component analysis-based strategy for Automatic Removal of Motion Artifact was used to denoise the MB data. Studies have shown ICA-AROMA outperforms other ICA-based denoising techniques such as FMRIB’s ICA-based X-noiseifier (FIX) (Griffanti et al., 2014; Salimi-Khorshidi et al., 2014). Dipasquale et al. (2017) evaluated ICA-AROMA in comparison to standard denoising, FIX, and ME-ICA for resting state fMRI[44]. They found ME-ICA performed best for multi-echo data while ICA-AROMA performed best for single echo data. Another advantage of ICA-AROMA is, unlike FIX, no training data is required (Dipasquale et al., 2017)[44]. While not the main goal of our study, we were able to show advanced denoising techniques, ICA-AROMA and ME-ICA outperformed standard denoising techniques. For example, mean *z*-scores and activation volumes were higher for the advanced denoising techniques (see Figure 3 and Tables 1, 3).

The voxelwise LME results showed MBME scans also had higher rCVR throughout much of the GM. This was confirmed via the ROI approach where rCVR was higher for MBME vs. MB for 12 of 17 GM ROIs. However, two ROIs showed decreased rCVR for MBME vs. MB. These ROIs corresponded to areas of the brain, including the orbital frontal gyrus and inferior temporal lobe, known for a high susceptibility-related signal dropout. In fact, the MB had artifactually high rCVR in these regions because low signal and high noise resulted in high PSC (Figure 4). Mean *z*-scores in these regions were lower for the MB data than for the MBME data (Figure 3), indicating poor signal quality and that the rCVR was artifactually high in these regions. The mean rCVRs for the MBME scans in these regions were more similar to the rest of the brain.

Heightened CVR was also seen in the cerebellum and visual cortex compared to the rest of the brain. One study has shown higher CVR in the cerebellum and occipital regions compared with the rest of the brain due to increased BOLD signal changes in those regions (Kastrup et al., 1999a). This area also corresponds to the vascular territory of the posterior inferior cerebral artery.

It is important to note higher rCVR does not necessarily indicate higher accuracy. In fact, rCVR depends on the region used for normalization. Here, mean GM CVR was used to normalize the CVR data. That region included the OFC and inferior temporal cortex, areas of significantly heightened CVR for the MB data. Therefore, the mean GM CVR may have been artifactually higher leading to lower CVR values. The goal of the rCVR analysis was to show the differences between the sequences in areas of high susceptibility. Therefore, these results indicate that the properties of the combined-echo data provide a more sensitive approach to detect rCVR values within difficult imaging domains such as the frontal orbital and inferior temporal areas (Figures 3, 4). This enhanced performance by the MBME sequence is also shown in Fernandez et al. (2017), where an ME EPI sequence outperformed the standard EPI sequence for both control and high-susceptibility-prone regions (such as the ventromedial prefrontal cortex, bilateral insula, and anterior cingulate cortex) for a fear conditioning task while using the same $T_{2}^{*}$-weighted echo combination approach (Fernandez et al., 2017). In future research, the MBME sequence could be applied to different tasks on those regions with high signal dropout, to test their cognitive or emotional regulatory roles in neurological and psychiatry conditions.

Very limited research has been done directly evaluating MBME sequences compared with MB sequences. In particular, no study has reported such a comparison by assessing the rCVR measurements. Different from the Cohen and Wang (2019) study, this study used a pure MB acquisition with a shorter TR of <1 s, in order to directly compare it with an MBME sequence. These findings align with other reports using ME and simultaneous multi-slice approaches that displayed better identification of BOLD components, a better ability to filter out artifacts, and gains in sensitivity (Boyacioglu et al., 2015; Olafsson et al., 2015).

Whole-brain normalized rCVR was used instead of absolute CVR for two main reasons. First, another BOLD fMRI CVR study recommended utilizing rCVR to understand regional distributions because it reduces intra-group variations by normalizing to the whole brain CVR (Yezhuvath et al., 2009). Second, since MBME uses a weighted combination of three echoes with TE ranging from 11 to 49 ms, the MB and MBME scans had different effective TEs. The BOLD PSC is known to increase with increasing TE (Fujita, 2001; Yacoub et al., 2003; Triantafyllou et al., 2011). Therefore, the CVR was normalized to provide a fair comparison between the scans.

One limitation of this study is that the effect of age and gender was not fully assessed; also, disease-related factors were not evaluated as only healthy subjects participated. It is important to note that in cases of physiological changes that have CVR effects involving the entire brain, absolute CVR would be reported. However, as this study emphasizes, rCVR data are useful in cases where the expected CVR changes are regional and can better reveal between-group differences over time. In addition, in order to complete a consistent evaluation, spatial resolution was matched for MB and MBME. It is important to understand that adjustments between TR and voxel size can be done for MB but that such adjustments for MBME are more limited due to the long echo-train length. However, future directions could take advantage of emerging gradient technology that involves increasing strength and slew rate to allow MBME to be more compatible with higher spatial resolutions (Foo et al., 2018). Finally, the FA was the same (60°) for the MBME and MB data despite differing TRs. The FA was set to the Ernst angle for the MBME, but should have been adjusted to the Ernst angle for the MB acquisition (52.7°) as well. This error only resulted in a signal intensity difference of approximately 1% (see Figure 1A).

Previous works have already revealed BH BOLD fMRI as a practical strategy for absolute CVR measurements that can provide group differences for healthy individuals (Urback et al., 2017). Further research is necessary to assess the applicability of BH MBME BOLD fMRI in patients with neurological diseases, especially in those with known vascular effects such as Alzheimer’s, epilepsy, Moyamoya, and traumatic brain injury.

## Conclusion

In conclusion, MBME and MB sequences were compared in terms of rCVR. The MBME approach enhanced the BH activation strength and volume as well as the rCVR repeatability and reliability, especially in the regions with high signal dropout observed in single echo imaging. This suggests that MBME EPI with appropriate processing is a useful option for obtaining reliable CVR measurements.

## Data Availability Statement

The original contributions presented in the study are included in the article/supplementary material, further inquiries can be directed to the corresponding author/s.

## Ethics Statement

The studies involving human participants were reviewed and approved by Medical College of Wisconsin/Froedtert Hospital Institutional Review Board. The patients/participants provided their written informed consent to participate in this study.

## Author Contributions

AC: conceptualization, methodology, software, investigation, data analysis, writing–original draft, and writing–revision. AJ and NV: data analysis and writing–original draft. BY and SB: conceptualization, resources, and writing–review and editing. BF: conceptualization, resources, writing–review and editing, and supervision. YW: conceptualization, resources, methodology, investigation, data analysis, writing–review and editing, and supervision. All authors contributed to the article and approved the submitted version.

## Conflict of Interest

BY, BF, and SB are employed by GE Healthcare. The remaining authors declare that the research was conducted in the absence of any commercial or financial relationships that could be construed as a potential conflict of interest.

## Footnotes

1 https://tedana.readthedocs.io/en/latest/
